# Analytical performance evaluation of a commercial next generation sequencing liquid biopsy platform using plasma ctDNA, reference standards, and synthetic serial dilution samples derived from normal plasma

**DOI:** 10.1186/s12885-020-07445-5

**Published:** 2020-10-01

**Authors:** Suman Verma, Mathew W. Moore, Rebecca Ringler, Abhisek Ghosal, Kyle Horvath, Theodore Naef, Sheri Anvari, Philip D. Cotter, Shelly Gunn

**Affiliations:** 1 ResearchDx, Inc., 5 Mason, Irvine, CA USA; 2 PacificDx Clinical Laboratory, 5 Mason, Irvine, CA USA

**Keywords:** ctDNA, Liquid biopsy, Tumor biomarkers, NGS

## Abstract

**Background:**

Circulating tumor (ct) DNA assays performed in clinical laboratories provide tumor biomarker testing support for biopharmaceutical clinical trials. Yet it is neither practical nor economically feasible for many of these clinical laboratories to internally develop their own liquid biopsy assay. Commercially available ctDNA kits are a potential solution for laboratories seeking to incorporate liquid biopsy into their test menus. However, the scarcity of characterized patient samples and cost of purchasing validation reference standards creates a barrier to entry. In the current study, we evaluated the analytical performance of the AVENIO ctDNA liquid biopsy platform (Roche Sequencing Solutions) for use in our clinical laboratory.

**Method:**

Intra-laboratory performance evaluation of AVENIO ctDNA Targeted, Expanded, and Surveillance kits (Research Use Only) was performed according to College of American Pathologists (CAP) guidelines for the validation of targeted next generation sequencing assays using purchased reference standards, de-identified human plasma cell-free (cf) DNA samples, and contrived samples derived from commercially purchased normal and cancer human plasma. All samples were sequenced at read depths relevant to clinical settings using the NextSeq High Output kit (Illumina).

**Results:**

At the clinically relevant read depth, Avenio ctDNA kits demonstrated 100% sensitivity in detecting single nucleotide variants (SNVs) at ≥0.5% allele frequency (AF) and 50% sensitivity in detecting SNVs at 0.1% AF using 20–40 ng sample input amount. The assay integrated seamlessly into our laboratory’s NGS workflow with input DNA mass, target allele frequency (TAF), multiplexing, and number of reads optimized to support a high-throughput assay appropriate for biopharmaceutical trials.

**Conclusions:**

Our study demonstrates that AVENIO ctDNA liquid biopsy platform provides a viable alternative for efficient incorporation of liquid biopsy assays into the clinical laboratory for detecting somatic alterations as low as 0.5%. Accurate detection of variants lower than 0.5% could potentially be achieved by deeper sequencing when clinically indicated and economically feasible.

## Background

Genomic analysis of tumor DNA is integral to diagnosis and treatment planning for many human malignancies, particularly non-small cell lung cancer (NSCLC) [1]. Yet, inaccessibility of tumor tissue and the patient’s underlying medical condition can complicate the use of core needle biopsies for retrieval of tumor DNA. In situations where tissue biopsy is contraindicated, the ctDNA component of cell free (cf) DNA can be accessed through routine phlebotomy and evaluated by molecular methods for detection of somatic (tumor specific) genomic alterations [2]. Deep sequencing analysis of circulating tumor (ct) DNA obtained by liquid biopsy provides a minimally invasive method for comprehensive evaluation of actionable tumor biomarkers [3–5]. Additionally, access to tumor DNA through the patient’s blood allows incorporation of prospectively-planned longitudinal biomarker monitoring into biopharmaceutical clinical trials as opposed to relying on the diagnostic biopsy to provide a static guide to the tumor mutation landscape [6, 7]. As an example, formalin fixed, paraffin embedded (FFPE) tumor DNA preserved in tissue blocks at the time of diagnosis may not be representative of targetable genomic alterations (such as EGFR T790M) developing weeks to months post-biopsy through tumor genome evolution [8].

Incorporation of liquid biopsy into a clinical trial protocol requires support of a clinical laboratory capable of providing high-throughput deep sequencing for ctDNA detection. Although applications for liquid biopsy are increasing, widespread adoption by clinical laboratories has been impeded by multiple factors including the need for ultralow detection limits, interference from sequencing artifacts at low allele frequencies, and the requirement for coverage of diverse mutation types with broad clinical applications [9, 10]. Given these design considerations and bioinformatic challenges, it is neither practical nor economically feasible for most clinical laboratories (even those already running NGS testing) to internally develop an in-house liquid biopsy assay. Use of commercially available ctDNA kits is an alternative strategy for laboratories seeking to incorporate liquid biopsy sample analysis into their test menus. For laboratories launching ctDNA testing with a commercial kit, critical considerations include the number of genes and types of variants represented by the pre-selected gene panel, sensitivity and specificity of the kit for detection of somatic mutations at low (< 1%) allele frequencies, ease of assay workflow, hands-on time, sample input requirements, and a robust bioinformatics pipeline that provides a clinically actionable variant report.

Liquid biopsy assays face unique technical challenges. The cfDNA is fragmented (~ 160 bp) and present at a very small quantity in patient samples (typically < 10 ng/ml plasma). In addition, tumor DNA (ctDNA) is a minute fraction of cfDNA, making it highly challenging to accurately detect rare variants (< 1%) from low ctDNA inputs. A number of bioinformatic solutions are now available for addressing the unique analysis challenges created by extremely low ctDNA levels in cancer patient plasma [11, 12]. Our laboratory chose to evaluate the AVENIO ctDNA liquid biopsy platform (Roche Sequencing Solutions, Pleasanton, CA) as a commercially available option to provide the laboratory with an end-to-end solution (DNA extraction to clinically actionable patient report) for testing liquid biopsy samples. AVENIO ctDNA analysis kits are based on cancer personalized profiling by deep sequencing (CAPP-Seq) [13] with options for Targeted, Expanded and Surveillance panels designed to interrogate clinically relevant mutations in 17, 77 or 197 genes (Supplementary Figure S1, S2 and S3), respectively [14]. In addition, the Avenio platform includes specialized bioinformatics analysis workflow which has integrated digital error suppression (iDES) system. iDES augments CAPP-Seq through in silico removal of stereotypical sequencing artifacts combined with molecular barcoding [13]. In the current study we evaluated the analytical performance characteristics of all three AVENIO kits using purchased reference standards, human plasma cfDNA samples, and contrived samples derived from normal human plasma.

## Methods

### Analytical evaluation

Intra-laboratory analytical evaluation and validation of the AVENIO ctDNA Analysis Kit was performed at Pacific Diagnostics (PacificDx, Irvine, CA) according to College of American Pathologists guidelines for validation of targeted next generation sequencing assays [15, 16]. Accuracy, precision, and limit of detection were evaluated in the following sample types: reference standards purchased from Horizon Discovery (Waterbeach, UK; *n* = 8, Multiplex I cfDNA Reference Standard Set) and SeraCare (*n* = 7) (Milford, MA; n = 7, Seraseq ctDNA Mutation Mix v2 and Seraseq ctDNA complete mutation mix); cfDNA extracted from normal plasma (*n* = 8) purchased from Biological Specialty Corporation (Colmar, PA); human plasma samples collected from various cancer individuals (*n* = 12) purchased from ProteoGenex, Inc. (Ingelwood, CA), synthetic serial dilution samples of “mutant” and wild type (WT) cfDNA derived from normal plasma (*n* = 4) produced at Pacific Diagnostics; and NA12878 (*n* = 1) as a negative control (Coriell, Camden NJ.) Reference Standards were chosen to represent all variation classes (SNVs, indels, CNAs and SVs) tested by the assay. Standards were engineered by the manufacturer (Horizon and Seracare) to contain representative mutations within human cancer cell lines with DNA fragmented to an average fragment length of 160 bp, resembling cfDNA extracted from plasma. Allelic frequency and copy number data provided by the manufacturer via Droplet Digital PCR (ddPCR) was used to perform concordance with data obtained from Avenio ctDNA analysis kits. In addition, 67 de-identified cfDNA samples at concentrations from 10 to 50 ng were evaluated for observance of QC metrics across all three panels (Targeted, Expanded, Surveillance.)

### Synthesis of serial dilution samples

Normal human plasma from de-identified donors was purchased from Biological specialty corporation (Allentown, PA). Multiple cfDNA replicates were extracted from each of the four normal human plasma samples. DNA libraries were created from each donor and sequenced in quadruplicate using the AVENIO ctDNA Expanded Kit (Roche Sequencing, Pleasanton, CA). An additional four normal human plasma specimens were extracted and sequenced in singlicate. Variants reported in the unfiltered data set of AVENIO software were used to identify 22 “mutations” present in one of the normal plasma samples at ~ 50% allele frequency, designated as the “mutant” sample. Selected mutations were identified in the following genes: *NTRK1*, *FGFR2*, *BRCA1*, *BRCA2*, *ALK*, *FGFR3*, *PDGFRA*, *CSF1R*, *PMS2*, *PTCH1* (Table 1). Three normal plasma samples lacking these 22 mutations were designated as the normal wild type (WT) and mixed to create a background normal sample. Serial dilutions were prepared by spiking the mutant into the WT with resultant expected allele frequencies of: 5.0, 1.0, 0.5, and 0.1%. Dilutions were made such that the total amount of cfDNA for each reaction was 12 ng.

**Table 1:** Synthetic serial dilution variants

Sr#	Gene Name	chr	Position	dbSNP ID	Coding change	Amino acid change	AF (%)
1	NTRK1	chr1	156876441	rs6334	c.1674G > A	p.Gln558Gln	43.7
2	FGFR2	chr10	121551357	rs755793	c.557 T > C	p.Met186Thr	48.6
3	BRCA2	chr13	32332343	rs766173	c.865A > C	p.Asn289His	43.7
4	BRCA2	chr13	32332843	rs1801439	c.1365A > G	p.Ser455Ser	41
5	BRCA2	chr13	32336584	rs1801499	c.2229 T > C	p.His743His	50.3
6	BRCA2	chr13	32337326	rs1799944	c.2971A > G	p.Asn991Asp	46.5
7	BRCA2	chr13	32355095	rs1799955	c.7242A > G	p.Ser2414Ser	46.1
8	BRCA2	chr13	32379413	rs11571769	c.8851G > A	p.Ala2951Thr	48.1
9	BRCA1	chr17	43071077	rs1799966;rs730880287	c.4900A > G	p.Ser1634Gly	46.7
10	BRCA1	chr17	43082453	rs1060915;rs397509161	c.4308 T > C	p.Ser1436Ser	47.9
11	BRCA1	chr17	43091983	rs16942	c.3548A > G	p.Lys1183Arg	46.3
12	BRCA1	chr17	43092418	rs16941	c.3113A > G	p.Glu1038Gly	49.3
13	BRCA1	chr17	43093220	rs16940	c.2311 T > C	p.Leu771Leu	47.8
14	BRCA1	chr17	43093449	rs1799949	c.2082C > T	p.Ser694Ser	45.7
15	ALK	chr2	29320870	rs35093491	c.1427 T > C	p.Val476Ala	48.4
16	FGFR3	chr4	1799815	rs3135868	c.445 + 3A > G	N/A	43.7
17	FGFR3	chr4	1801524	rs2305181	c.603 T > C	p.Ile201Ile	49.5
18	PDGFRA	chr4	54263911	rs2229307	c.612 T > C	p.Asn204Asn	46.3
19	PDGFRA	chr4	54267559	rs4358459	c.939 T > G	p.Gly313Gly	51.2
20	CSF1R	chr5	150080792	rs41287102	c.282C > T	p.Ser94Ser	46.1
21	PMS2	chr7	5982995	rs140788589	c.2007-4G > A	N/A	45.7
22	PTCH1	chr9	95476097	rs1805155	c.1665 T > C	p.Asn555Asn	47.1

Variant calls were generated by AVENIO Oncology Analysis Server (OAS). Twenty-two mutations were identified at ~ 50% allele frequency in one of the normal plasma sample. This sample was designated as mutant sample. The sample was serially diluted in the background of pooled cfDNA generated from normal plasma samples lacking these mutations, to generate plasma cfDNA samples mutations ranging from 5 to 0.1% AF

### Isolation of cfDNA from plasma using the Avenio cfDNA extraction kit

Whole blood was collected in K2-EDTA tubes. Plasma was separated by double centrifugation at 1500 x g for 10 min with brakes off within 4 h. of collection. Plasma was stored frozen at − 70-80 °C until cfDNA isolation.

In preparation for DNA isolation, 2–5 ml of plasma was thawed, centrifuged at 1800 x g for 5 min at room temperature and transferred to a conical tube. After addition and incubation with Proteinase K, DNA Paraffin Binding Buffer, and isopropanol, samples were transferred to a High Pure Extender Assembly (HPEA) unit. The HPEA unit comes pre-assembled as a 50 ml tube containing a High Pure Extender to hold the plasma and a Filter Tube to bind the cfDNA. Following centrifugation for 5 min at 3270 x g, the Filter Tube was removed from the HPEA and placed into a collection tube. The cfDNA was then washed and eluted from the detached filter tube. Following isolation of the purified cfDNA, samples were either stored at 2–8 °C or used immediately for DNA library preparation. After extraction, cfDNA concentration was quantified using the Qubit dsDNA High Sensitivity Assay (ThermoFisher Scientific, Waltham, MA) and recorded along with the calculated isolated DNA mass. Additionally, sample quality was confirmed using the High Sensitivity DNA Kit on the Agilent 2100 Bioanalyzer.

### Preparation of sequencing libraries using the Avenio kit

Sequencing libraries with unique sample indexes were prepared using the AVENIO cfDNA Library Prep sub-Kit (Roche) according to the manufacturer’s instructions. In brief, 10 μl of unique Sample Adapter was added to each sample followed by overnight incubation at 16 °C. Following post-ligation cleanup using AVENIO cleanup beads, the library molecules were amplified using the AVENIO Pre-Enrichment PCR Master Mix reagents and primers. Following post-PCR cleanup, library size and quantity were verified by High Sensitivity DNA Kit on the Agilent Bioanalyzer and Qubit. After library QC, samples were either stored at − 15 to − 20 °C or moved immediately to the Library Enrichment using the AVENIO ctDNA Enrichment Kit, one of the three AVENIO Panels (Targeted, Expanded, or Surveillance), and the AVENIO Post-Hybridization sub-Kits according to the manufacturer’s instructions. Post-hybridization washes were followed by a second PCR amplification step in preparation for sequencing. Following post-PCR cleanup, samples were stored at − 15 to − 20 °C or moved quantified for sequencing. Library concentration was assessed using the Qubit dsDNA High Sensitivity Assay followed by Bioanalyzer High Sensitivity DNA kit on Bioanalyzer to determine the size of each library. Region tables were set from 200 to 1000 bp and library sizes were recorded. Library quantification was confirmed using absolute qPCR-based quantification of libraries containing Illumina P5 and sequences using the KAPA Universal Library Quantification kit (KapaBiosystems, Wilmington, MA).

### Preparation of sequencing libraries using the TruSight oncology 500 ctDNA kit

cfDNA was extracted from 6 ml of plasma samples utilizing the QIAamp circulating Nucleic Acid Kit (QIAGEN, Cat No./ID: 55114) according to kit instructions. Libraries were constructed using a minimum 30 ng of cfDNA measured by capillary electrophoresis method (75-300 bp range) as per the manufacturer’s instructions for the TruSight Oncology 500 ctDNA Kit (Illumina, 20,012,860). Indexed pre-capture libraries were enriched for specific targeted regions covered by the TruSight Oncology 500 ctDNA kit by two rounds of hybridization, streptavidin bead capture and clean up. The enriched libraries were amplified, purified with sample purification beads and normalized with normalization beads prior to sequencing. Samples were sequenced on a NovaSeq 6000 instrument using either a S2 300 cycle Reagent Kit (8 samples/run) or a S4 300 cycle Reagent Kit (6 samples/lane or 24 samples/flow cell) in conjunction with a NovaSeq Xp 4-Lane Kit ((Illumina, 20,012,866 and 20,021,665). Data analysis was performed utilizing the DRAGEN TruSight Oncology 500 ctDNA Analysis Software with Illumina DRAGEN server v3.

### DNA sequencing and data analysis of the Avenio data

The addition of unique sample adapters during library prep enabled multiplexed sequencing of up to 11 samples per sequencing run for the AVENIO Expanded and Surveillance kits. Higher multiplexing (up to 16 samples per Nextseq High output run) was achieved for the AVENIO Targeted kit, which has a smaller panel. Library pools were prepared by combining an equal mass of each library and sequenced using the 300 cycle NextSeq 500/550 High Output kit v2.5 on the NextSeq 500/550 (Illumina, San Diego CA). Data was analyzed with the AVENIO ctDNA Analysis Software (version 1.0.0 & 2.0.0).

## Results

### Assay QC metrics

**Fig. 1:**
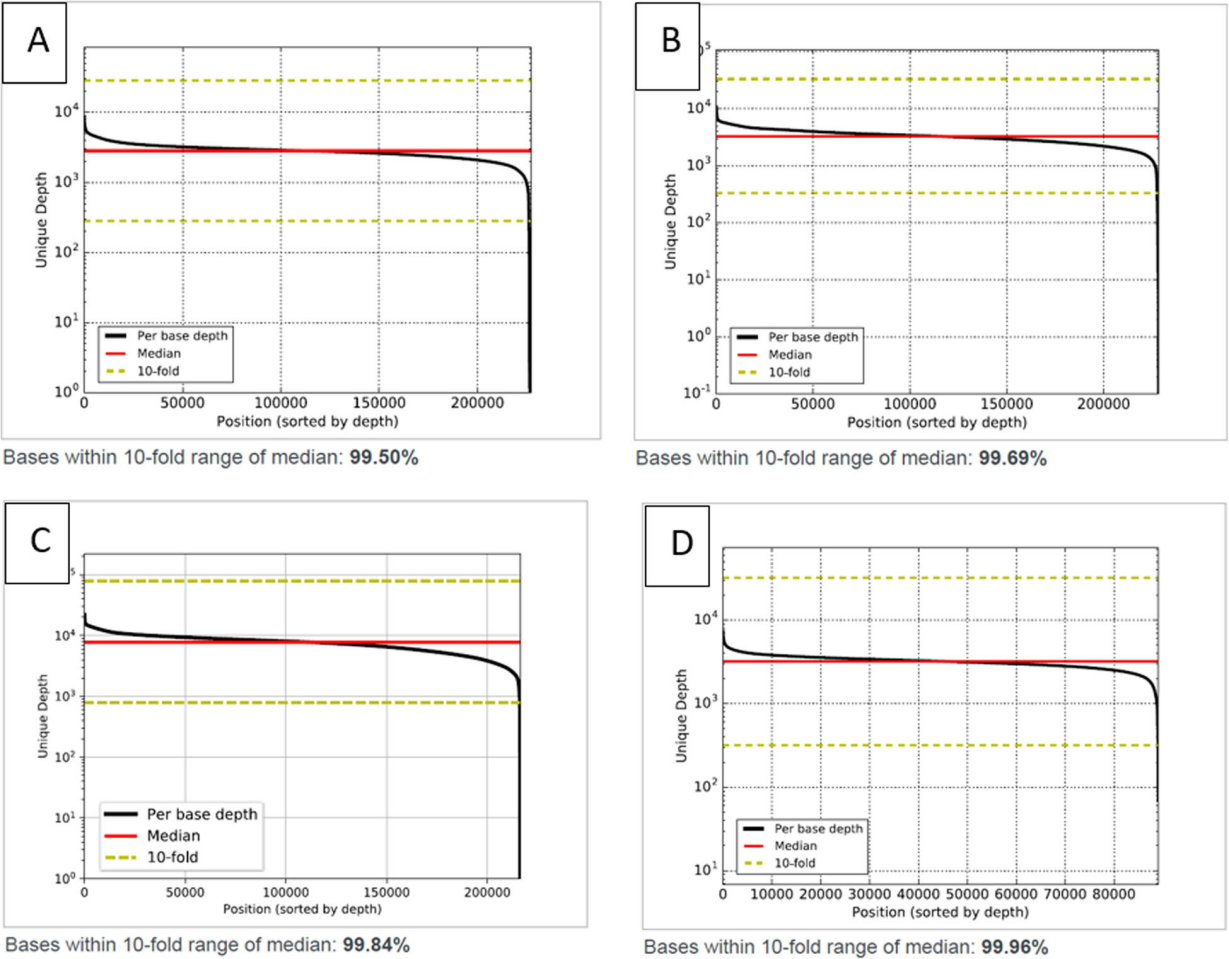
Example coverage uniformity obtained on all three panels. **a**. Plasma sample tested using Expanded panel **b**. ctDNA reference standard tested using Expanded panel **c**. Plasma sample tested using Surveillance panel **d**. Plasma sample tested using Targeted panel

Assay performance QC was tested using 67 human plasma and reference standards across all three AVENIO ctDNA analysis kits [Expanded (28), Targeted (29), and Surveillance [10]]. Sample inputs across the entire recommended range (10–50 ng) were tested. The sequencing depth per sample was determined by the panel used to ensure adequate coverage: an average of 30 million paired end reads were obtained for the AVENIO Expanded and Surveillance kit samples and 20 million paired end reads were obtained for AVENIO Targeted kit samples (Table 2 and Supplementary Table S1). All three kits obtained an average of 70% on-target reads. Because the unique coverage is a function of DNA input, the unique median coverage obtained was dependent on sample input: 50 ng sample input resulted in 7000-8000X unique coverage and 10 ng sample input resulted 1000-1100X unique coverage. All three kits had low error rate (1–3 X10^− 5^) and uniform coverage (98–100% of the targeted regions were covered within 10-fold of the median coverage) (Fig. 1). All three kits across all 67 samples displayed >500x unique depth coverage even in lowest 5’th percentile of target region (Table 2).

**Table 2:** Sample QC Metrics. Data in Table was generated by AVENIO OAS

Panel		Input DNA Mass (ng)	# Read Pairs	Sequencing Depth Median	Unique Depth Median	Unique Depth 5’th Percentile	Unique Depth 95’th Percentile	Error Rate	On-Target Rate	Bases Within 10-fold Range of Median	N
AVENIO Expanded Kit	Minimum	10	23,167,961	8146	1046	846	3248	0.000004	54%	98%	28
	Maximum	50	38,724,317	15,142	7423	3258	14,192	0.000064	78%	99%	28
	Average	31	29,508,308	10,054	4632	2182	9686	0.000017	69%	99%	28
AVENIO Targeted Kit	Minimum	11	14,972,469	15,076	1121	721	1855	0.000005	70%	100%	29
	Maximum	50	25,743,805	24,697	8028	5900	17,389	0.000021	81%	100%	29
	Average	28	20,645,107	19,999	3423	2493	5757	0.000010	77%	100%	29
AVENIO Surveillance Kit	Minimum	20	25,469,334	5451	2626	1485	4460	0.000003	26%	100%	10
	Maximum	50	38,331,741	15,096	7857	3336	11,878	0.000072	79%	100%	10
	Average	29	33,150,021	11,223	5041	2393	7432	0.000034	65%	100%	10

### Accuracy, sensitivity, specificity and limit of detection

Assay sensitivity and specificity were tested for AVENIO Expanded, Targeted and Surveillance panels by comparing expected results against experimental results from well characterized reference standards and four blended human normal plasma samples (236 variants across 14 samples). All three panels displayed 100% sensitivity in detecting Single Nucleotide Variants (SNVs) and Indels at 0.5% allele frequency (AF) in samples with an input range from 20 ng–40 ng (68 variants). The AVENIO Surveillance panel displayed 100% sensitivity for detecting SNVs/Indels at 0.5% AF, 72% sensitivity at 0.25% AF, and 28% sensitivity at 0.1% AF (18 variants at each AF) at 20 ng sample input amount (Table 3 and Supplementary Table S3). The sensitivity for SNVs/Indels in the AVENIO Expanded kit were 100% at 1% AF, 100% at 0.5% AF, and 50% at 0.1% AF with 40 ng sample input (8 variants at each AF) As expected, the sensitivity was reduced to 100% at 1% AF, 82% at 0.5% AF, and 29% at 0.1% AF (28 variants at each AF) with 15 ng input (Table 3 and Supplementary Table S2). Sensitivity for detecting SNV and INDELs by Targeted panel was 100% at 0.5%AF at 20 ng input and it was reduced to 93% at 10 ng input (Table 3 and Supplementary Table 4). All three panels displayed 100% sensitivity in detecting all fusions and amplifications down to 2.5% AF except CD74-ROS1 fusion. CD74-ROS1 fusion was captured and sequenced by both Targeted and Expanded panel (Table 4 and data not shown). However, it was not called by the Avenio software at any allele frequency tested. This could possibly be due to synthetic fusion created in the reference standard that was limited to short low complexity region of the CD74 gene and was filtered out in Avenio software. In future, it will be of interest to test this fusion with other reference standards or plasma samples.

**Table 3:** Avenio ctDNA kit sensitivity for SNV and INDEL

Sensitivity at AF% (SNV/Indel)								
Kit	Sample Input (ng)	Multiplexing on NextSeq	Number of variants	AF 2.5%	AF 1%	AF 0.5%	AF 0.25%	AF 0.1%
Expanded	40	11 High	8	N/A	100%	100%	N/A	50%
Expanded	15	11 High	28	N/A	100%	82%	N/A	29%
Surveillance	20	11 High	18	N/A	100%	100%	72%	28%
Targeted	20	16 High	14	100%	N/A	100%	N/A	N/A
Targeted	10	16 High	14	100%	N/A	93%	N/A	N/A

**Table 4:** Avenio ctDNA kit sensitivity for Fusions and amplifications

	Sample ID	Sample Name	Sample Type	Sample input amount (ng)	Type	Gene	Variant	Expected Result	Experimental Result	Analytical Sensitivity
Expanded	HD786	5% structural ref. std.	Reference Std	40	AMPLIFICATION	MET	N/A	AMP	AMP	100%
					FUSION	ROS1	SLC34A2/ROS1	5.6%	SLC34A2 ROS1	
					FUSION	RET	CCDC6/RET	5.6%	RET CCDC6	
	“HD787”	2.5% structural ref. std.	Reference Std	40	AMPLIFICATION	MET	N/A	AMP	AMP	100%
					FUSION	ROS1	SLC34A2/ROS1	2.8%	SLC34A2 ROS1	
					FUSION	RET	CCDC6/RET	2.8%	RET CCDC6	
	“HD788”	1% structural ref. std.	Reference Std	40	AMPLIFICATION	MET	N/A	AMP	AMP	67%
					FUSION	ROS1	SLC34A2/ROS1	1.1%	SLC34A2-ROS1	
					FUSION	RET	CCDC6/RET	1.1%	ND	
Surveillance	0710–140	Seracare 1% ref. std	Reference Std	20	FUSION	ALK	TPR-ALK	1.00%	TPR-ALK	100%
					FUSION	RET	NCOA4-RET	1.00%	NCOA4-RET	
	0710–141	Seracare 0.5% ref. std	Reference Std	20	FUSION	ALK	TPR-ALK	0.50%	ND	50%
					FUSION	RET	NCOA4-RET	0.50%	NCOA4-RET	
Targeted	0710–0529	Seracare 2.5% ref. std	Reference Std	20	FUSION	ALK	EML4-ALKv1	2.5%	ALK;EML4	80%
					FUSION	RET	NCOA4-RET	2.5%	RET;NCOA4	
					FUSION	ROS1	CD74-ROS1**	2.5%	ND**	
					AMPLIFICATION	ERBB2	ERBB2	2.5%	AMP	
					AMPLIFICATION	MET	MET	2.5%	AMP	
	0710–0531	Seracare 0.5% ref. std	Reference Std	20	FUSION	ALK	EML4-ALKv1	0.5%	ND	0%
					FUSION	RET	NCOA4-RET	0.5%	ND	
					FUSION	ROS1	CD74-ROS1	0.5%	ND	
					AMPLIFICATION	ERBB2	ERBB2	0.5%	ND	
					AMPLIFICATION	MET	MET	0.5%	ND	
	0710–0529	Seracare 2.5% ref. std	Reference Std	10	FUSION	ALK	EML4-ALKv1	2.5%	ALK;EML4	60%
					FUSION	RET	NCOA4-RET	2.5%	RET;NCOA4	
					FUSION	ROS1	CD74-ROS1**	2.5%	ND**	
					AMPLIFICATION	ERBB2	ERBB2	2.5%	ND	
					AMPLIFICATION	MET	MET	2.5%	AMP	
	0710–0531	Seracare 0.5% ref. std	Reference Std	10	FUSION	ALK	EML4-ALKv1	0.5%	ND	0%
					FUSION	RET	NCOA4-RET	0.5%	ND	
					FUSION	ROS1	CD74-ROS1	0.5%	ND	
					AMPLIFICATION	ERBB2	ERBB2	0.5%	ND	
					AMPLIFICATION	MET	MET	0.5%	ND	

**The fusion was captured by the panel but it was not called by Avenio software

Additionally, the Expanded panel was tested for specificity using one wild type reference standard from Horizon (HD776), one well characterized HAPMAP cell line (NA12878), and eight normal human plasma sample. Specificity for the Surveillance panel was tested using one wild type reference standard and NA12878. Specificity for the Targeted panel was tested using a Seracare reference standard (0.5 and 2.5% AF). Specificity for SNV and INDEL for each panel was limited to variants in loci of interest for each panel. All three panels displayed very high specificity for detecting SNV ≥0.5% AF, INDELs, fusions and amplifications (100%). Limited number of SNV were detected below 0.5%. Two plasma samples displayed germline variants (Table 5). A PMS2, p.Lys651Arg variant was detected in four out of eight plasma samples tested indicating high prevalence of this mutation in general population. Overall, all three panels displayed > 99.7% specificity in detecting SNV and INDEL within the loci of interest region and 100% specificity in detection of fusions and amplifications (Table 5).

**Table 5:** AVENIO ctDNA kit specificity

		# of mutations Detected	Gene Name	Cosmic ID	Mutation	AA change	TYPE	AF (%)	Loci of interest	Analytical Specificity
Expanded (527 SNV + 75 INDEL present in LOI)	NA12878	0	N/A	N/A	N/A	N/A	N/A	N/A	N/A	100%
	LS 2396760B	2	ALK	COSM28498	c.3512 T > A	p.Ile1171Asnp.Ile131Asn	SNV	0.05%	Present	99.8%
			PMS2	COSM5095519	c.1634A > G	p.Lys651Arg	SNV	0.40%	Absent	
	LS 2396761B	1	MSH2	COSM1408245	c.502 T > C	p.Ser168Pro	SNV	48.4%	Absent	100%
	LS 8833056D	1	PMS2	COSM5095519	c.1634A > G	p.Lys651Arg	SNV	0.63%	Absent	100%
	LS 8833058D	1	PMS2	COSM5095519	c.1634A > G	p.Lys651Arg	SNV	0.88%	Absent	100%
	LS 8835298	0	N/A	N/A	N/A	N/A	N/A	N/A	N/A	100%
	LS 8835299	2	PMS2	COSM5095519	c.1634A > G	p.Lys651Arg	SNV	0.87%	Absent	99.8%
			DPYD	COSM5095519	c.1679 T > G	p.Ile560Ser	SNV	48.8%	Present	
	LS 8835301	0	N/A	N/A	N/A	N/A	N/A	N/A	N/A	100%
	LS 8835303	1	PTEN	COSM23626	c.962_963insA	p.Asn323fs	SNV	0.13%	Present	99.8%
Surveillance (470 SNV + 66 INDEL present in LOI)	NA12878	1	TP53	COSM28498	c.726C > A	p.Cys242*	SNV	0.43%	Absent	100%
Targeted (252 SNV + 66 INDEL present in LOI)	0710–0529 (10 ng)	3	TP53	COSM1640832	c.734G > A	p.Gly245Asp	SNV	0.35%	Absent	99.7%
			ALK	COSM4751917	c.875G > A	p.Arg292His	SNV	0.38%	Absent	
			MET	N/A	c.2942-10C > G	N/A	SNV	0.03%	Present	
	0710–0529 (20 ng)	1	TP53	COSM1640832	c.734G > A	p.Gly245Asp	SNV	0.37%	Absent	100%
	0710–0531 (10 ng)	2	TP53	COSM1640832	c.734G > A	p.Gly245Asp	SNV	0.33%	Absent	100%
			ERBB2	COSM6002535	c.1777C > A	p.Pro593Thr	SNV	0.17%	Absent	
	0710–0531 (20 ng)	1	TP53	COSM1640832	c.734G > A	p.Gly245Asp	SNV	0.33%	Absent	100%

Accuracy of the Avenio assay was further determined by testing plasma samples collected from 12 cancer patients (breast cancer, colorectal cancer, prostate cancer and NSCLC; 3 each) at various stages of cancer (stage IIB-IV) using the Avenio Expanded kit and the TruSight500 (TSO500) ctDNA kit (Illumina, Inc.). The TruSight500 ctDNA kit is a liquid biopsy kit from Illumina that can detect alterations at low allele frequencies (≥0.5% AF) in cfDNA samples. The TruSight and Avenio Expanded kits have many genes that are covered in common, making TruSight a suitable kit to compare data obtained from the Avenio kit. Eight ml of plasma from each cancer specimen was purchased from a commercial vendor (ProteoGenex, Inc.) and was split between the Avenio Expanded kit and the TSO500ctDNA kit for testing (2 ml and 6 ml respectively). cfDNA destined for the Avenio assay was extracted using the cfDNA extraction kit provided with the Avenio assay. cfDNA destined for the TSO500 kit was extracted using the QIAamp circulating nucleic acid kit (Qiagen, Inc.) 10–50 ng cfDNA input was used for the Avenio assay except for two samples where 9.0 ng input was used. As per the manufacturer’s recommendation, a minimum 30 ng input was used for the TSO500 ctDNA assay. Two samples did not meet sample input requirements for the TSO 500 ctDNA kit and were not tested. Eight of the 12 cancer patients detected alterations (SNV and CNV) in the frequently mutated cancer genes (3/3 CRC, 2/3 breast cancer, 1/3 prostate cancer and 2/3 NSCLC) using the Avenio assay. Six of eight positive samples were also tested using the TSO500 ctDNA kit and the same alterations were detected at similar allele frequency range in both assays. Similarly, four of four negative samples also did not detect any alteration in the Avenio intersected region in the TruSight500 assay, indicating 100% concordance between the Avenio and the TSO500 ctDNA assays (Table 6).

**Table 6:** Accuracy for detecting alteration in human cancer samples

Sr#	ID	Cancer Type	Input DNA Mass (ng)	Isolated DNA mass (ng)	On-Target%	Unique Median Depth	Alteration Type Detected	Gene	Coding Change	Protein Change	Avenio (AF%)	TSO500 ctDNA (AF%)	Concordant (Y/N)
1	025587P	CRC (Stage IIIC)	9	9.7	63%	2609	SNV	PIK3CA	c.1633G > A	p.Glu545Lys	0.31%	0.47%	Y
							SNV	ERBB2	c.2524G > A;c.2524G > A;c.2479G > A	p.Val842Ile;p.Val842Ile;p.Val827Ile	0.32%	0.26%	Y
2	025831P	CRC (Stage IIIC)	15.7	20.41	70%	2725	SNV	PIK3CA	c.1633G > A	p.Glu545Lys	0.24%	Not Tested^a^	N/A
							SNV	KRAS	c.182A > T; c.182A > T	p.Gln61Leu;p.Gln61Leu	0.37%		
							SNV	APC	c.4031C > G	p.Ser1344*	0.50%		
3	025622P	CRC (Stage IVA)	30.05	39.07	66%	2004	SNV	KRAS	c.35G > A; c.35G > A	p.Gly12Asp;p.Gly12Asp	12.35%	15.34%	Y
							SNV	APC	c.4012C > T	p.Gln1338*	20.45%	25.69%	Y
							CNV	EGFR	N/A	N/A	AMP	AMP	Y
4	019391P	Breast Cancer (stage IIB)	10.25	13.33	71%	2557	None	N/A	N/A	N/A	N/A	None	Y
5	019396P	Breast Cancer (Stage IIB)	10	13	69%	2272	SNV	DDR2	c.2515C > T	p.Arg839Cys	0.27%	Not Tested^a^	N/A
6	019388P	Breast Cancer (Stage IIB)	13.15	17.1	70%	2876	SNV	PIK3CA	c.1624G > A	p.Glu542Lys	0.44%	0.83%	Y
7	045931P	NSCLC (Stage IIIB)	16.25	21.13	72%	2836	SNV	TP53	c.892G > T;c.892G > T;c.892G > T;c.859G > T	p.Glu298*;p.Glu298*;p.Glu298*;p.Glu287*	23.47%	28.00%	Y
							CNV	EGFR	N/A	N/A	AMP	AMP	Y
8	045903P	NSCLC(Stage IIB)	12.1		66%	2138	SNV	PTEN	c.1027-A > G	N/A	2.04%	2.62%	Y
9	045911P	NSCLC (Stage IIB)	15.1	19.63	70%	2648	None	N/A	N/A	N/A	N/A	None	Y
10	163277P	Prostate Cancer (Stage III)	16.4	21.32	71%	2905	SNV	TP53	c.832C > T; c.832C > T; c.832C > T; c.799C > T	p.Pro278Ser;p.Pro278Ser;p.Pro278Ser;p.Pro267Ser	1.28%	2.01%	Y
11	163445P	Prostate Cancer (Stage IV)	15.75	20.48	66%	2801	None	N/A	N/A	N/A	N/A	None	Y
12	163477P	Prostate Cancer (Stage III)	9	9.5	65%	1689	None	N/A	N/A	N/A	N/A	None	Y

Comparative study was performed between Avenio ctDNA Expanded kit and TruSight500 ctDNA kit using 12 cancer specimens collected from patients diagnosed with Breast cancer (stage IIB), Colorectal Cancer (Stage III-IV), NSCLC (stage II-III) and Prostate Cancer (Stage III-IV)

^a^Two of the 12 samples did not give enough cfDNA for testing using TruSight500 ctDNA kit

### Precision

Assay precision was tested by with five reference standards with 0–5% AF and four normal plasma samples. Each precision sample was subjected to three library preparations, sequencing runs, and data analyses (Table 7). Library preparations for repeatability (Run 1 and Run 2) were prepared on the same day, used the same lot of library preparation reagents, and were run on a single NextSeq run. Library preparations for reproducibility (Run 3) were performed on a different day from the first two runs by a distinct operator and sequenced on a separate NextSeq run. Assay concordance was 100% for SNVs and indels at allele frequencies ≥0.5%. As expected, the variant frequency variability (%CV) was higher for variants with lower allele frequencies (≤ 1%).

**Table 7:** Avenio Expanded ctDNA analysis kit precision

Sample ID	Sample Name	Type	Gene	Variant	Expected Result	Rep 1	Rep 2	Rep 3	Average	%CV	Average %CV
HD777	5% ref. std	SNV	NRAS	p.Ala59Thr	6.3%	4.66%	5.52%	5.33%	5.17%	7.13%	6.74%
		SNV	NRAS	p.Gln61Lys	6.3%	5.58%	4.98%	6.53%	5.70%	11.20%	
		SNV	KRAS	p.Gly12Asp	6.3%	6.30%	5.53%	5.25%	5.69%	7.80%	
		SNV	PIK3CA	p.Glu545Lys	6.3%	6.08%	5.80%	5.67%	5.85%	2.92%	
		SNV	EGFR	p.Thr790Met	5.0%	4.17%	4.72%	4.93%	4.61%	6.96%	
		SNV	EGFR	p.Leu858Arg	5.0%	4.80%	4.42%	5.23%	4.82%	6.87%	
		INDEL	EGFR	p.V769-D770insAlaSerVal	5.0%	4.20%	4.80%	4.40%	4.47%	5.58%	
		INDEL	EGFR	p.Glu746_Ala750del	5.0%	4.90%	4.30%	4.50%	4.57%	5.46%	
HD778	1% ref. std	SNV	NRAS	p.Ala59Thr	1.3%	0.92%	0.76%	1.04%	0.91%	12.65%	18.17%
		SNV	NRAS	p.Gln61Lys	1.3%	1.04%	1.18%	1.07%	1.10%	5.49%	
		SNV	KRAS	p.Gly12Asp	1.3%	1.06%	1.10%	1.00%	1.05%	3.90%	
		SNV	PIK3CA	p.Glu545Lys	1.3%	1.06%	1.24%	0.98%	1.09%	9.94%	
		SNV	EGFR	p.Thr790Met	1.0%	0.29%	0.52%	0.83%	0.55%	40.47%	
		SNV	EGFR	p.Leu858Arg	1.0%	0.68%	0.76%	1.12%	0.85%	22.43%	
		INDEL	EGFR	V769-D770insAlaSerVal	1.0%	0.80%	1.00%	1.10%	0.97%	12.90%	
		INDEL	EGFR	p.Glu746_Ala750del	1.0%	1.00%	0.50%	0.46%	0.65%	37.60%	
“HD780”	0.5% ref. std	SNV	NRAS	p.Ala59Thr	0.6%	0.36%	0.66%	0.43%	0.48%	26.51%	24.59%
		SNV	NRAS	p.Gln61Lys	0.6%	0.91%	0.56%	0.37%	0.61%	36.47%	
		SNV	KRAS	p.Gly12Asp	0.6%	0.52%	0.62%	0.80%	0.65%	17.92%	
		SNV	PIK3CA	p.Glu545Lys	0.6%	0.55%	0.54%	0.70%	0.60%	12.27%	
		SNV	EGFR	p.Thr790Met	0.5%	0.45%	0.40%	0.56%	0.47%	14.22%	
		SNV	EGFR	p.Leu858Arg	0.5%	0.46%	0.56%	0.38%	0.47%	15.78%	
		INDEL	EGFR	V769-D770insAlaSerVal	0.5%	0.10%	0.23%	0.32%	0.22%	41.68%	
		INDEL	EGFR	p.Glu746_Ala750del	0.5%	0.16%	0.15%	0.29%	0.20%	31.89%	
HD779	0.1% ref. std	SNV	NRAS	p.Ala59Thr	0.1%	ND	ND	ND	NA	NA	N/A
		SNV	NRAS	p.Gln61Lys	0.1%	0.14%	0.16%	ND	0.15%	6.67%	
		SNV	KRAS	p.Gly12Asp	0.1%	0.24%	0.26%	ND	0.25%	4.00%	
		SNV	PIK3CA	p.Glu545Lys	0.1%	ND	ND	ND	NA	NA	
		SNV	EGFR	p.Thr790Met	0.1%	ND	ND	ND	NA	NA	
		SNV	EGFR	p.Leu858Arg	0.1%	0.06%	ND	0.18%	0.12%	50.00%	
		INDEL	EGFR	V769-D770insAlaSerVal	0.1%	0.12%	ND	ND	0.12%	0.00%	
		INDEL	EGFR	p.Glu746_Ala750del	0.1%	ND	ND	ND	N/A	N/A	
HD777	WT ref. std	SNV	NRAS	p.Ala59Thr	0.0%	0.0%	0.0%	0.0%	N/A	N/A	N/A
		SNV	NRAS	p.Gln61Lys	0.0%	0.0%	0.0%	0.0%			
		SNV	KRAS	p.Gly12Asp	0.0%	0.0%	0.0%	0.0%			
		SNV	PIK3CA	p.Glu545Lys	0.0%	0.0%	0.0%	0.0%			
		SNV	EGFR	p.Thr790Met	0.0%	0.0%	0.0%	0.0%			
		SNV	EGFR	p.Leu858Arg	0.0%	0.0%	0.0%	0.0%			
		INDEL	EGFR	V769-D770insAlaSerVal	0.0%	0.0%	0.0%	0.0%			
		INDEL	EGFR	p.Glu746_Ala750del	0.0%	0.0%	0.0%	0.0%			
LS2396760B	Plasma	SNV	KDR	p.Gly770Val	0.0%	0.09%	ND	ND	N/A	N/A	N/A
		SNV	PMS2	p.Lys651Arg	0.0%	0.49%	0.40%	0.52%			
		INDEL	VHL	p.Phe148fs	0.0%	0.12%	ND	ND			
		SNV	ALK	p.Ile131Asn	0.0%	ND	0.05%	ND			
		INDEL	KIT	p.Val559del	0.0%	ND	ND	0.11%			
		SNV/INDEL	All	All = 564	0.0%	ND	ND	ND			
LS2396761	Plasma	SNV	MSH2	p.Ser168Pro	0.0%	51.0%	48.4%	49.3%	N/A	N/A	N/A
		SNV	KDR	p.Arg347Cys	0.0%	0.18%	ND	ND			
		SNV/INDEL	All	All = 567	0.0%	ND	ND	ND			
LS8833056	Plasma	SNV	PMS2	p.Lys651Arg	0.0%	0.63%	ND	0.37%	N/A	N/A	N/A
		SNV	VHL	p.Phe148fs	0.0%	ND	ND	0.13%			
		SNV/INDEL	All	All = 567	0.0%	ND	ND	ND			
LS8833058	Plasma	SNV	KRAS	p.Gly12Arg	0.0%	0.11%	ND	ND	N/A	N/A	N/A
		SNV	PMS2	p.Lys651Arg	0.0%	0.88%	ND	0.51%			
		SNV/INDEL	All	All = 567	0.0%	ND	ND	ND			

### Assay linearity

**Fig. 2:**
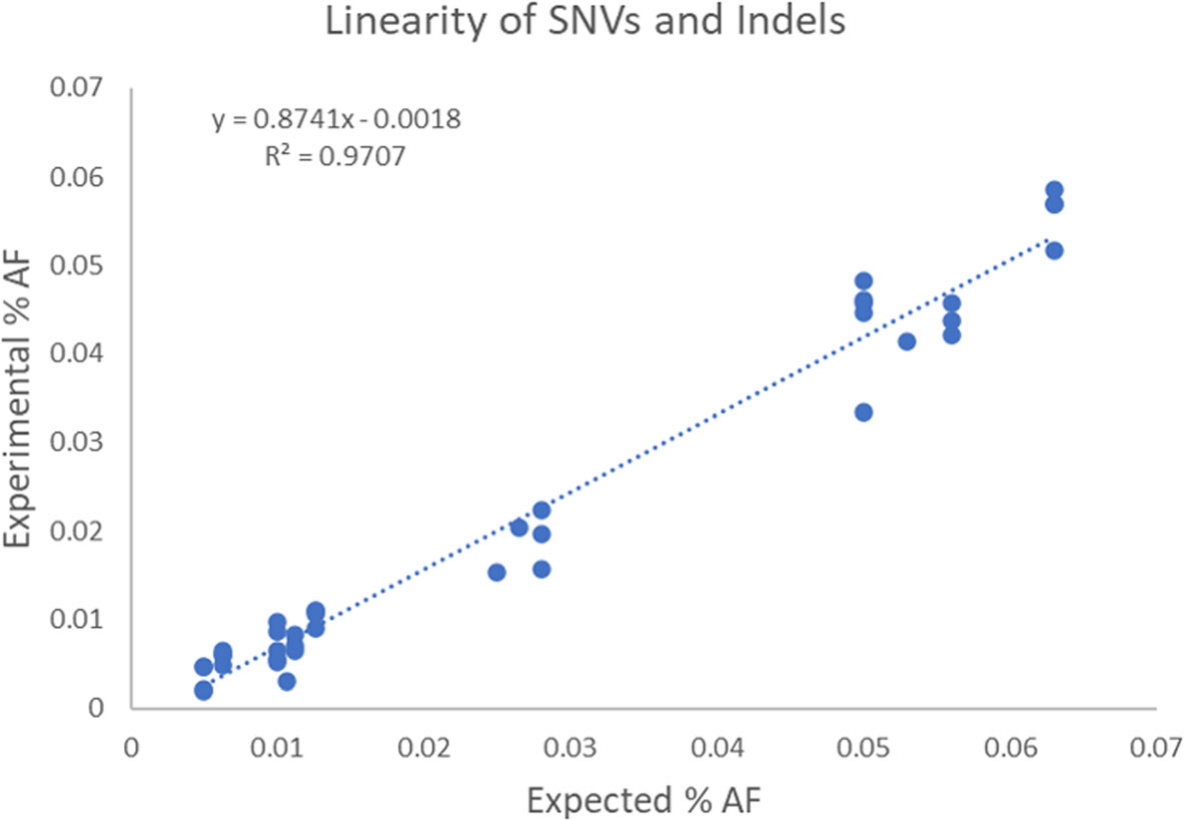
Linearity of SNVs and Indels – Experimental versus Expected AF values for the AVENIO Expanded Panel data

To assess linearity of the AVENIO Expanded panel for variants at allele frequencies below 5%, the reported allele frequency values for SNVs and indels in the reference standards and normal human plasma samples (with frequencies ranging from 0.5–6%; 47 total data points) were plotted against the expected allele frequency values and fitted by linear regression. The correlation was high (R2 = 0.979), suggesting quantitative accuracy at low variant frequencies (Fig. 2).

### Reportable range

The reportable range of the AVENIO ctDNA Expanded, Targeted, and Surveillance panels, is defined as the fraction of targeted genomic regions for which calls of acceptable quality can be generated [17]. The Expanded Panel contains 77 genes, the Targeted Panel contains 17 genes and the Surveillance Panel contains 197 genes, including those currently in the US National Comprehensive Cancer Network (NCCN) Guidelines. The total panel size for the Expanded kit is 192 kb, the Targeted kit is 81 Kb and the Surveillance kit is 198 kb. All three panels include all four mutation classes – SNVs, INDELs, fusions and CNA. The assay reports SNV in all regions interrogated by the assay (Supplementary Figure S1, S2 and S3). INDELs are limited to variants in a pre-specified list of positions, referred to as “Loci of Interest”, except for EGFR exon 19 long deletions, EGFR exon 20 long insertions, and MET long insertions, which are not restricted to a pre-defined set of Indels (Supplementary Table S5 and S6). CNV is limited to MET, ERBB2 and EGFR genes in all three panels. The fusions are tested for 6 gene targets (ALK, ROS1, RET, NTRK1, FGFR2 and FGFR3) in the Expanded panel and 3 gene targets in the Targeted and Surveillance panels (ALK, ROS1 and RET). All three panels displayed very high uniformity of coverage (≥99% bases within 10-fold of median; Fig. 1) and displayed >500x unique depth coverage even in the lowest 5’th percentile of target regions covered by the panel (Table 2). No low coverage or drop out regions were detected during this validation. Although not called by the Avenio reporting software, the CD74-ROS1 fusion was manually detected in BAM file. The CD74 region of the fusion is in a GA-rich, low complexity region (as defined by Repeat Masker), which suggests the fusion call is being filtered out with current version of software (v2.0.0). As such, the ROS1-CD74 fusion will not be included in reportable range for Avenio assay for this software version.

## Discussion

**Fig. 3:**
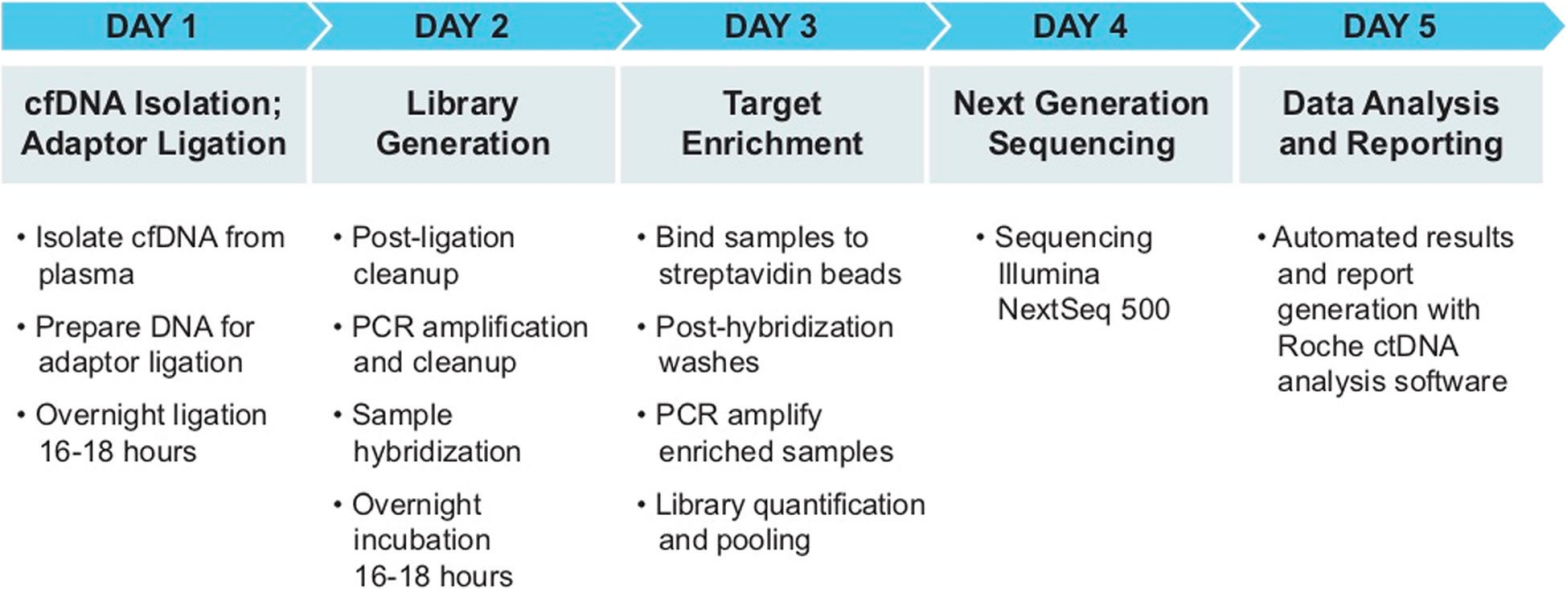
Avenio workflow

In our study, we evaluated the performance and accuracy of a commercially available ctDNA liquid biopsy platform. The AVENIO assay integrated seamlessly into our laboratory’s existing NGS workflow with an average sample-to-report TAT of 5 working days (Fig. 3). Multiple stopping points are included within the protocol where samples can be frozen to accommodate a variety of laboratory schedules, including those without weekend staff coverage. As our laboratory was already performing NGS using hybrid capture for library preparation, there was no interruption to established protocols or need for new equipment to incorporate AVENIO assays into the daily testing routine. The iDES insert and index barcodes incorporated into DNA strands during library preparation create unique identifiers (UIDs) used for sample multiplexing [13]. Technicians performing the assay noted little or no difference in procedures between FFPE (data not shown) and ctDNA DNA kits.

In our hands all three AVENIO kits displayed very high on-target rate (~ 70%; Supplementary Table 1) and median unique depth (~4000x unique coverage using 30 ng input) on human plasma samples. Unique depth is a critical measure of performance in liquid biopsy assays as it indicates how many initial cfDNA molecules were retained throughout the library prep and sequencing process and ultimately dictates the assay sensitivity. Similarly, the panel displayed very high precision (< 25%CV) in detection of variants ≤0.5% AF. The increased CV at lower allele frequency is expected due to Poisson variance when performing library prep on very few mutant ctDNA molecules. Higher variance at low allele frequency can result in false negative and/or false positive results for variants < 0.5% AF.

We chose to perform in-depth validation of AVENIO ctDNA Expanded panel for intra-laboratory accuracy studies since the clinically informative genetic alterations targeted by the panel are suitable for biopharma clinical trial support. The 77 genes targeted by the panel represent 567 known hotspot tumor variants, including specific alterations in genes such as *EGFR*, *KRAS,* and *BRAF* linked to eligibility for on-label targeted therapies [14, 18]. Additional genes and gene regions allow for discovery of off-label actionable biomarkers. The Expanded Panel also incorporates recurrent genetic alterations from the CAPP-Seq selector library allowing identification of a unique cancer personalized profile (CAPP) that can be monitored throughout the course of a patient’s disease [9]. Additionally, advantages to hybrid capture-based enrichment strategies, in contrast to amplification-based assays, are well documented and include minimal allele dropout, deeper uniform coverage, and higher sensitivity [19]. A recent retrospective matched tissue-plasma analysis from NSCLC subjects using the AVENIO Surveillance hybrid capture strategy reported tissue-plasma concordance to be positively associated with tumor size and cancer stage [20].

As an additional evaluation of the AVENIO assay we compared accuracy and performance with Illumina’s TSO500 liquid biopsy panel for somatic mutations in intersected regions. We found that both panels displayed similar performance characteristics. However, TSO500 panel required higher number of sequencing reads due to its larger panel size (500 genes) and required higher cfDNA input (30 ng) making it less economical and clinically feasible in comparison to Avenio assay. A similar study was recently reported by Lam, et al. in which the AVENIO platform was compared to Qiagen’s QIAseq Human Comprehensive Cancer panel for panel coverage of clinically relevant variants and overall sequencing performance [21]. Detailed results from both comparison studies reveal strengths and shortcomings unique to each of these different assays. Overall, any of these NGS assay provide much larger amount of clinically relevant information than traditional targeted ddPCR assays, however NGS assays can be more expensive than PCR assays. Based on the strength and weakness of each assay clinical laboratories should make informed decisions about which panel(s) and platforms are best suited to their specific needs.

As the intended use of the panel is for routine monitoring of tumor biomarkers, we chose economical sample multiplexing and a higher TAF of ≥0.5% in order to maximize the number of samples (*n* = 11) per NextSeq run. Optimal performance was observed using 10–50 ng of cfDNA input mass and 30 million paired-end reads with deduplicated coverage averaging above 4000 reads per base. Although some laboratories are seeking to identify variants at lower TAFs using alternative methodologies, we found the commercial kit performance for multiplexed samples to be most robust at TAFs of ≥0.5%. This finding is supported by other studies demonstrating higher correlation with tissue biopsy results at AFs in the range of 1.0% [20, 22]. In clinical scenarios where it is biologically indicated for small tumors or low levels of residual disease, the AVENIO Expanded panel can be run in “High Output” mode with as few as 1–3 samples to achieve near 90% sensitivity at TAF of 0.05% [17].

## Conclusions

The AVENIO liquid biopsy platform provides a user friendly, accurate solution for incorporating ctDNA analysis into the workflow of an NGS laboratory. In the “moderate output” mode tested in this study, performance is most robust at TAFs ≥0.5%, suitable for an intended use in clinical trials of late stage cancers where a higher ctDNA content is expected in the plasma due to larger and/or metastatic tumors. When biologically indicated and economically feasible, future studies of “high output” mode for lower allele frequencies (≤ 0.1%), reflective of small tumors and minimal residual disease, will be performed by our laboratory. With increasing adoption of ctDNA analysis in the clinical laboratory, we envision incorporation of routine tumor biomarker monitoring for patients enrolled in oncology clinical trials.

## Supplementary information

**Additional file 1:** **Supplementary Figure S1.** Targeted regions covered by Avenio Expanded Panel.

**Additional file 2:** **Supplementary Figure S2.** Targeted regions covered by Avenio Surveillance Panel.

**Additional file 3:** **Supplementary Figure S3.** Targeted regions covered by Avenio Targeted Panel

**Additional file 4:** **Supplementary Table S1.** Sample QC Metrics across three panels [Targeted, Expanded, Surveillance]

**Additional file 5:** **Supplementary Table S2.** AVENIO Expanded ctDNA analysis kit sensitivity (SNV/INDEL)

**Additional file 6:** **Supplementary Table S3.** Avenio Surveillance ctDNA kit sensitivity (SNV/INDEL)

**Additional file 7:** **Supplementary Table S4.** Avenio Targeted ctDNA kit sensitivity (SNV/INDEL)

**Additional file 8:** **Supplementary Table S5.** INDEL Loci of interest for Avenio Expanded kit

**Additional file 9:** **Supplementary Table S6.** INDEL Loci of interest for Avenio Targeted and Surveillance kit

## Data Availability

The datasets generated and/or analyzed during the current study are available in the NCBI Sequence Read Archive (SRA), project ID PRJNA66392; study ID SRP282644; https://www.ncbi.nlm.nih.gov/sra?term=SRP282644

## Abbreviations

cfDNA: cell free DNA
ctDNA: circulating tumor DNA
SNV: Single nucleotide variant
AF: Allele frequency
IDES: Integrated digital error suppression
CAPP-Seq: Cancer personalized profiling by deep sequencing
UIDs: Unique identifiers
TAF: Target allele frequency
