# Supplementary figures and images for: Analytical performance evaluation of a commercial next generation sequencing liquid biopsy platform using plasma ctDNA, reference standards, and synthetic serial dilution samples derived from normal plasma
Source: BMC Cancer. 2020 Oct 1;20:945. doi: 10.1186/s12885-020-07445-5 (PMC7528227; doi:10.1186/s12885-020-07445-5)

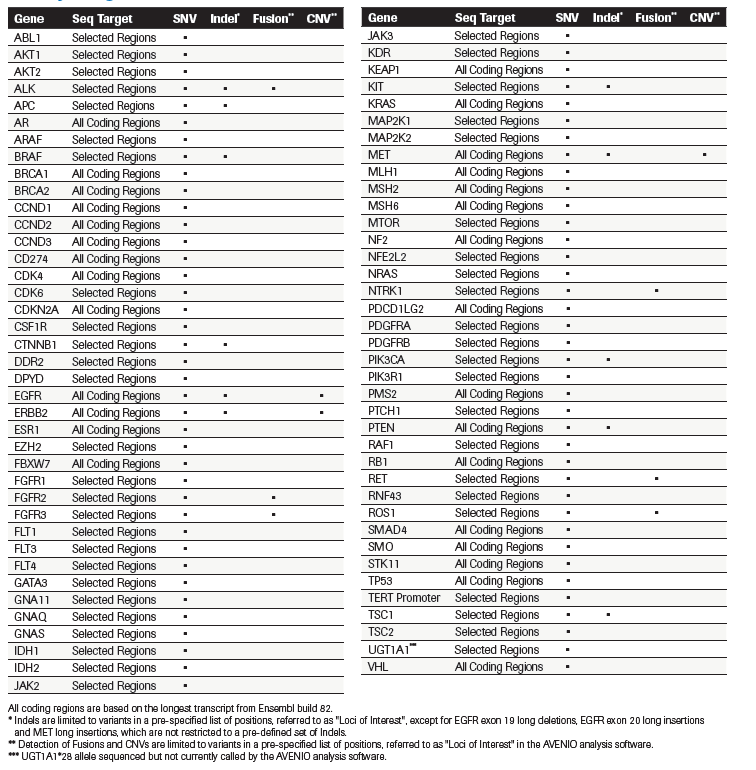

Supplement: Supplementary file 1 — Additional file 1: Supplementary Figure S1. Targeted regions covered by Avenio Expanded Panel. [file 12885_2020_7445_MOESM1_ESM.tif]

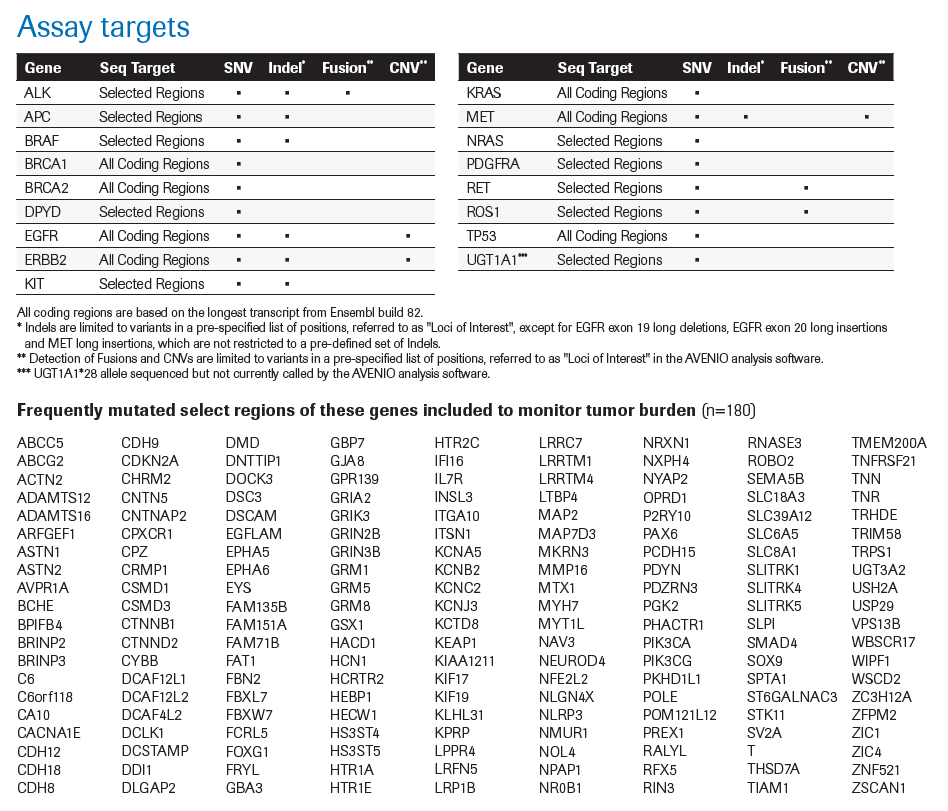

Supplement: Supplementary file 2 — Additional file 2: Supplementary Figure S2. Targeted regions covered by Avenio Surveillance Panel. [file 12885_2020_7445_MOESM2_ESM.tif]

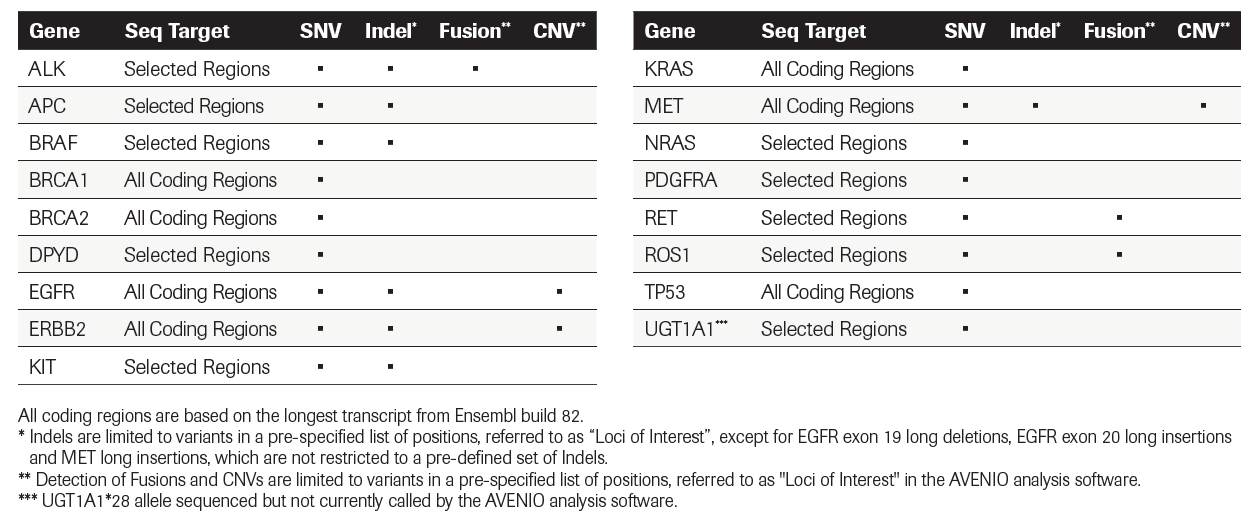

Supplement: Supplementary file 3 — Additional file 3: Supplementary Figure S3. Targeted regions covered by Avenio Targeted Panel [file 12885_2020_7445_MOESM3_ESM.tif]
