# Supplementary material for: Analytical performance evaluation of a commercial next generation sequencing liquid biopsy platform using plasma ctDNA, reference standards, and synthetic serial dilution samples derived from normal plasma
Source: BMC Cancer. 2020 Oct 1;20:945. doi: 10.1186/s12885-020-07445-5 (PMC7528227; doi:10.1186/s12885-020-07445-5)
Supplement: Supplementary file 4 — Additional file 4: Supplementary Table S1. Sample QC Metrics across three panels [Targeted, Expanded, Surveillance] [file 12885_2020_7445_MOESM4_ESM.docx]

**Supplementary Table S1:** Sample QC Metrics across three panels [Targeted, Expanded, Surveillance]

| Sr # | Sample ID | Sample Type | Panel | cfDNA (ng) | Mapped Reads | # Read Pairs (M) | Sequencing Depth Median | Unique Depth Median | On-Target Rate | Bases Within 10-fold Range of Median |
| --- | --- | --- | --- | --- | --- | --- | --- | --- | --- | --- |
| 1 | 190006 | Plasma | Expanded | 39 | 83% | 29 | 9643 | 5673 | 74% | 99% |
| 2 | 190008 | Plasma | Expanded | 50 | 83% | 27 | 9844 | 2114 | 78% | 99% |
| 3 | 190010 | Plasma | Expanded | 50 | 83% | 28 | 8531 | 5575 | 70% | 99% |
| 4 | 190011 | Plasma | Expanded | 31 | 87% | 30 | 10340 | 5978 | 77% | 99% |
| 5 | 190013 | Plasma | Expanded | 41 | 86% | 29 | 9556 | 5967 | 74% | 99% |
| 6 | 190014 | Plasma | Expanded | 35 | 88% | 31 | 10764 | 6405 | 76% | 99% |
| 7 | 190017 | Plasma | Expanded | 45 | 88% | 34 | 11422 | 6630 | 75% | 99% |
| 8 | 190019 | Plasma | Expanded | 50 | 90% | 34 | 11971 | 7423 | 77% | 98% |
| 9 | 190026 | Plasma | Expanded | 50 | 82% | 28 | 9138 | 5842 | 75% | 99% |
| 10 | 190027 | Plasma | Expanded | 50 | 98% | 30 | 10656 | 6606 | 72% | 99% |
| 11 | 190043 | Plasma | Expanded | 30 | 94% | 29 | 9984 | 5967 | 67% | 99% |
| 12 | 190052 | Plasma | Expanded | 30 | 98% | 27 | 8893 | 5030 | 63% | 99% |
| 13 | 190053 | Plasma | Expanded | 30 | 94% | 29 | 9607 | 3468 | 67% | 99% |
| 14 | 190062 | Plasma | Expanded | 30 | 98% | 25 | 8889 | 5169 | 66% | 99% |
| 15 | 190065 | Plasma | Expanded | 30 | 92% | 32 | 10790 | 5967 | 65% | 99% |
| 16 | 190069 | Plasma | Expanded | 30 | 96% | 27 | 9518 | 5220 | 67% | 99% |
| 17 | 190070 | Plasma | Expanded | 28 | 96% | 27 | 9374 | 5159 | 66% | 99% |
| 18 | 190071 | Plasma | Expanded | 26 | 91% | 28 | 9414 | 4924 | 68% | 99% |
| 19 | 190073 | Plasma | Expanded | 29 | 98% | 23 | 8146 | 5041 | 64% | 99% |
| 20 | 190074 | Plasma | Expanded | 28 | 98% | 26 | 9296 | 5388 | 64% | 99% |
| 21 | 190023 | Plasma | Expanded | 49 | 83% | 33 | 10590 | 6583 | 73% | 99% |
| 22 | 190004 | Plasma | Expanded | 30 | 93% | 29 | 11130 | 1641 | 71% | 99% |
| 23 | 190002 | Plasma | Expanded | 15 | 86% | 30 | 10650 | 1046 | 77% | 99% |
| 24 | 190035 | Plasma | Expanded | 10 | 92% | 32 | 10819 | 2229 | 63% | 99% |
| 25 | 190036 | Plasma | Expanded | 10 | 95% | 39 | 15142 | 2019 | 75% | 99% |
| 26 | 190038 | Plasma | Expanded | 10 | 90% | 31 | 8797 | 2458 | 54% | 99% |
| 27 | 190050 | Plasma | Expanded | 10 | 91% | 32 | 10002 | 2069 | 59% | 99% |
| 28 | 190063 | Plasma | Expanded | 10 | 90% | 29 | 8596 | 2106 | 55% | 99% |
| 29 | 195115 | Plasma | Targeted | 35 | 86% | 25 | 24696.5 | 5686 | 79% | 100% |
| 30 | 195125 | Plasma | Targeted | 30 | 84% | 19 | 18416 | 3144 | 78% | 100% |
| 31 | 195129 | Plasma | Targeted | 30 | 84% | 19 | 17697 | 3543 | 75% | 100% |
| 32 | 195130 | Plasma | Targeted | 45 | 87% | 24 | 22632 | 5299 | 76% | 100% |
| 33 | 195147 | Plasma | Targeted | 50 | 89% | 22 | 20272 | 8028 | 70% | 100% |
| 34 | 195148 | Plasma | Targeted | 35 | 84% | 20 | 18664 | 3700 | 77% | 100% |
| 35 | 195149 | Plasma | Targeted | 36 | 85% | 23 | 21313 | 4097 | 76% | 100% |
| 36 | 195150 | Plasma | Targeted | 25 | 88% | 24 | 24004 | 3696 | 80% | 100% |
| 37 | 195151 | Plasma | Targeted | 50 | 97% | 18 | 18915.5 | 4701 | 72% | 100% |
| 38 | 195152 | Plasma | Targeted | 49 | 84% | 23 | 21323 | 4401 | 78% | 100% |
| 39 | 195157 | Plasma | Targeted | 28 | 83% | 20 | 18836 | 2885 | 75% | 100% |
| 40 | 195159 | Plasma | Targeted | 45 | 85% | 20 | 19303 | 2338 | 79% | 100% |
| 41 | 195161 | Plasma | Targeted | 45 | 87% | 26 | 22899 | 4829 | 73% | 100% |
| 42 | 195124 | Plasma | Targeted | 12 | 88% | 21 | 21605 | 2747 | 81% | 100% |
| 43 | 195126 | Plasma | Targeted | 21 | 87% | 20 | 20765 | 2585 | 80% | 100% |
| 44 | 195127 | Plasma | Targeted | 19 | 87% | 18 | 17856 | 2323 | 75% | 100% |
| 45 | 195131 | Plasma | Targeted | 14 | 84% | 21 | 20421 | 3344 | 77% | 100% |
| 46 | 195134 | Plasma | Targeted | 14 | 85% | 18 | 17341 | 2551 | 80% | 100% |
| 47 | 195136 | Plasma | Targeted | 18 | 88% | 23 | 23279 | 3201 | 80% | 100% |
| 48 | 195140 | Plasma | Targeted | 25 | 83% | 19 | 17515 | 3818 | 77% | 100% |
| 49 | 195141 | Plasma | Targeted | 12 | 86% | 20 | 19667.5 | 2775 | 79% | 100% |
| 50 | 195142 | Plasma | Targeted | 22 | 84% | 24 | 21635.5 | 4517 | 73% | 100% |
| 51 | 195146 | Plasma | Targeted | 25 | 91% | 21 | 21506 | 3442 | 75% | 100% |
| 52 | 195153 | Plasma | Targeted | 11 | 84% | 17 | 15987.5 | 1626 | 78% | 100% |
| 53 | 195154 | Plasma | Targeted | 21 | 86% | 15 | 15076 | 1121 | 80% | 100% |
| 54 | 195155 | Plasma | Targeted | 20 | 96% | 17 | 18428.5 | 1829 | 78% | 100% |
| 55 | 195158 | Plasma | Targeted | 21 | 83% | 17 | 16451 | 2008 | 77% | 100% |
| 56 | 195160 | Plasma | Targeted | 18 | 90% | 23 | 24244 | 2349 | 81% | 100% |
| 57 | 195162 | Plasma | Targeted | 25 | 83% | 20 | 19212 | 2692 | 76% | 100% |
| 58 | 200001 | Plasma | Surveillance | 28 | 85% | 25 | 10231 | 3368 | 78% | 100% |
| 59 | 200002 | Plasma | Surveillance | 41 | 85% | 33 | 13000 | 5669 | 78% | 100% |
| 60 | 200003 | Plasma | Surveillance | 50 | 88% | 38 | 15096 | 7857 | 77% | 100% |
| 61 | 200004 | Plasma | Surveillance | 50 | 83% | 29 | 11222 | 5801 | 77% | 100% |
| 62 | 200005 | Plasma | Surveillance | 29 | 81% | 28 | 10739 | 4223 | 79% | 100% |
| 63 | 200006 | Plasma | Surveillance | 20 | 94% | 29 | 12094 | 2626 | 79% | 100% |
| 64 | 200007 | Plasma | Surveillance | 20 | 94% | 36 | 13532 | 6168 | 64% | 100% |
| 65 | 200008 | Plasma | Surveillance | 20 | 91% | 38 | 5451 | 3687 | 26% | 100% |
| 66 | 200009 | Plasma | Surveillance | 20 | 93% | 36 | 10224 | 5213 | 51% | 100% |
| 67 | 200010 | Plasma | Surveillance | 20 | 94% | 36 | 11308 | 5754 | 53% | 100% |
