# Supplementary material for: Analytical performance evaluation of a commercial next generation sequencing liquid biopsy platform using plasma ctDNA, reference standards, and synthetic serial dilution samples derived from normal plasma
Source: BMC Cancer. 2020 Oct 1;20:945. doi: 10.1186/s12885-020-07445-5 (PMC7528227; doi:10.1186/s12885-020-07445-5)
Supplement: Supplementary file 5 — Additional file 5: Supplementary Table S2. AVENIO Expanded ctDNA analysis kit sensitivity (SNV/INDEL) [file 12885_2020_7445_MOESM5_ESM.docx]

**Supplementary Table S2:** AVENIO Expanded ctDNA analysis kit sensitivity (SNV/INDEL)

|  | | | | | AF 1% mix | | AF 0.5% mix | | AF 0.1% mix | |
| --- | --- | --- | --- | --- | --- | --- | --- | --- | --- | --- |
| Sample Type | Sample input amount (ng) | Gene ID | Variant | Type | Expected  Result | Experimental  Result | Expected  Result | Experimental  Result | Expected  Result | Experimental  Result |
| Reference Std | 40 | NRAS | p.Ala59Thr | SNV | 1.3% | 0.92% | 0.65% | 0.36% | 0.13% | ND |
|  |  | NRAS | p.Gln61Lys | SNV | 1.3% | 1.04% | 0.65% | 0.91% | 0.13% | 0.14% |
|  |  |  |  |  |  |  |  |  |  |  |
|  |  | KRAS | p.Gly12Asp | SNV | 1.3% | 1.06% | 0.65% | 0.52% | 0.13% | 0.24% |
|  |  | PIK3CA | p.Glu545Lys | SNV | 1.3% | 1.06% | 0.65% | 0.55% | 0.13% | ND |
|  |  | EGFR | p.Thr790Met | SNV | 1.0% | 0.29% | 0.50% | 0.45% | 0.10% | ND |
|  |  | EGFR | p.Leu858Arg | SNV | 1.0% | 0.68% | 0.50% | 0.46% | 0.10% | 0.06% |
|  |  | EGFR | V769-D770insAlaSerVal | INDEL | 1.0% | 0.80% | 0.50% | 0.10% | 0.10% | 0.12% |
|  |  | EGFR | p.Glu746_Ala750del | INDEL | 1.0% | 1.00% | 0.50% | 0.16% | 0.10% | ND |
| Contrived normal plasma mixture | 15 | PMS2 | p.Lys651Arg p.Lys545Arg | SNV | 0.6% | 0.23% | 0.50% | 0.61% | 0.50% | 0.60% |
|  |  | MTOR | p.Leu2303Leu | SNV | 2.0% | 2.20% | 1.00% | 0.70% | 0.20% | ND |
|  |  | MTOR | p.Ser1851Ser | SNV | 2.0% | 1.48% | 1.00% | 0.97% | 0.20% | ND |
|  |  | NTRK1 | p.Gln558Gln | SNV | 0.9% | 0.94% | 0.44% | ND | 0.09% | ND |
|  |  | FGFR2 | p.Met186Thr | SNV | 1.0% | 0.84% | 0.51% | 0.32% | 0.10% | 0.38% |
|  |  | BRCA2 | p.Asn289His | SNV | 0.9% | 0.42% | 0.46% | 0.28% | 0.09% | ND |
|  |  | BRCA2 | p.Ser455Ser | SNV | 0.9% | 0.84% | 0.43% | ND | 0.09% | 0.21% |
|  |  | BRCA2 | p.His743His | SNV | 1.0% | 0.67% | 0.48% | 0.26% | 0.10% | ND |
|  |  | BRCA2 | p.Asn991Asp | SNV | 0.9% | 0.81% | 0.47% | 0.38% | 0.09% | ND |
|  |  | BRCA2 | p.Ser2414Ser | SNV | 0.9% | 1.61% | 0.47% | 0.55% | 0.09% | ND |
|  |  | BRCA2 | p.Ala2951Thr | SNV | 0.9% | 0.86% | 0.47% | 0.75% | 0.09% | ND |
|  |  | BRCA1 | p.Ser1634Gly | SNV | 0.9% | 0.87% | 0.47% | 0.65% | 0.09% | ND |
|  |  | BRCA1 | p.Ser1436Ser | SNV | 1.0% | 1.03% | 0.48% | 0.72% | 0.10% | ND |
|  |  | BRCA1 | p.Lys1183Arg | SNV | 0.9% | 0.92% | 0.47% | ND | 0.09% | ND |
|  |  | BRCA1 | p.Glu1038Gly | SNV | 1.0% | 0.27% | 0.48% | 0.51% | 0.10% | 0.27% |
|  |  | BRCA1 | p.Leu771Leu | SNV | 0.9% | 0.65% | 0.46% | 0.62% | 0.09% | 0.18% |
|  |  | BRCA1 | p.Ser694Ser | SNV | 0.9% | 1.13% | 0.47% | 0.75% | 0.09% | ND |
|  |  | ALK | p.Val476Ala | SNV | 1.0% | 0.85% | 0.49% | 0.32% | 0.10% | ND |
|  |  | FGFR3 | c.445+3A>G | SNV | 0.9% | 0.94% | 0.43% | 0.22% | 0.09% | ND |
|  |  | FGFR3 | p.Ile201Ile | SNV | 0.9% | 1.04% | 0.46% | 0.41% | 0.09% | ND |
|  |  | PDGFRA | p.Asn204Asn | SNV | 0.9% | 0.90% | 0.46% | 0.32% | 0.09% | ND |
|  |  | PDGFRA | p.Gly313Gly | SNV | 1.0% | 0.78% | 0.49% | 0.30% | 0.10% | ND |
|  |  | CSF1R | p.Ser94Ser | SNV | 0.9% | 1.47% | 0.46% | ND | 0.09% | ND |
|  |  | PMS2 | c.2007-4G>A | SNV | 0.9% | 1.36% | 0.45% | ND | 0.09% | ND |
|  |  | PTCH1 | p.Asn555Asn | SNV | 1.0% | 0.99% | 0.48% | 0.48% | 0.10% | ND |
|  |  | GNAQ | p.Tyr101* | SNV | 0.80% | 0.72% | 0.80% | 0.85% | N/A | N/A |
|  |  | GNAQ | p.Thr96Ser | SNV | 0.80% | 0.72% | 0.80% | 1.00% | N/A | N/A |
|  |  | GNAQ | p.Met59Leu | SNV | 0.80% | 0.38% | 0.80% | 0.77% | N/A | N/A |
