# Supplementary material for: Analytical performance evaluation of a commercial next generation sequencing liquid biopsy platform using plasma ctDNA, reference standards, and synthetic serial dilution samples derived from normal plasma
Source: BMC Cancer. 2020 Oct 1;20:945. doi: 10.1186/s12885-020-07445-5 (PMC7528227; doi:10.1186/s12885-020-07445-5)
Supplement: Supplementary file 6 — Additional file 6: Supplementary Table S3. Avenio Surveillance ctDNA kit sensitivity (SNV/INDEL) [file 12885_2020_7445_MOESM6_ESM.docx]

**Supplementary Table S3:** Avenio Surveillance ctDNA kit sensitivity (SNV/INDEL)

|  |  |  |  |  | **AF 1% mix** | | **AF 0.5% mix** | | **AF 0.25% mix** | | **AF 0.1% mix** | |
| --- | --- | --- | --- | --- | --- | --- | --- | --- | --- | --- | --- | --- |
| Sample Type | Sample input amount (ng) | GeneID | Variant | Type | Expected  Result | Experimental  Result | Expected  Result | Experimental  Result | Expected  Result | Experimental  Result | Expected  Result | Experimental  Result |
| Reference Std | 20 | APC | p.R1450* | SNV | 0.87% | 0.79% | 0.50% | 0.59% | 0.33% | 0.18% | 0.18% | ND |
|  |  | BRAF | p.V600E | SNV | 1.05% | 0.85% | 0.54% | 0.45% | 0.26% | 0.23% | 0.14% | ND |
|  |  | CTNNB1 | p.T41A | SNV | 1.33% | 0.91% | 0.63% | 0.44% | 0.36% | 0.27% | 0.17% | ND |
|  |  | EGFR | p.E746_A750delELREA | Indel | 1.13% | 1.30% | 0.56% | 0.34% | 0.34% | 0.23% | 0.18% | ND |
|  |  | EGFR | p.D770_N771insG | Indel | 1.16% | 1.10% | 0.54% | 0.55% | 0.32% | 0.31% | 0.12% | ND |
|  |  | EGFR | p.L858R | SNV | 1.23% | 1.15% | 0.63% | 0.41% | 0.30% | 0.26% | 0.18% | ND |
|  |  | EGFR | p.T790M | SNV | 0.13% | 0.45% | 0.53% | 0.24% | 0.32% | 0.22% | 0.17% | ND |
|  |  | ERBB2 | p.A775_G776insYVMA | Indel | 0.98% | 0.30% | 0.53% | 0.17% | 0.27% | 0.11% | 0.14% | 0.13% |
|  |  | KIT | p.D816V | SNV | 1.11% | 0.92% | 0.60% | 0.38% | 0.32% | 0.19% | 0.13% | ND |
|  |  | KRAS | p.G12D | SNV | 0.98% | 1.12% | 0.53% | 0.56% | 0.28% | 0.31% | 0.15% | 0.13% |
|  |  | NRAS/CSDE1 | p.Q61R | SNV | 1.25% | 1.05% | 0.54% | 0.54% | 0.38% | 0.44% | 0.21% | 0.09% |
|  |  | PDGFRA | p.D842V | SNV | 1.14% | 0.74% | 0.53% | 0.43% | 0.26% | 0.29% | 0.14% | 0.16% |
|  |  | PIK3CA | p.E545K | SNV | 0.92% | 0.83% | 0.45% | 0.27% | 0.24% | ND | 0.13% | ND |
|  |  | PIK3CA | p.H1047R | SNV | 1.11% | 0.72% | 0.47% | 0.24% | 0.23% | ND | 0.13% | ND |
|  |  | RET | p.M918T | SNV | 1.07% | 0.65% | 0.55% | 0.38% | 0.28% | 0.13% | 0.14% | 0.14% |
|  |  | TP53 | p.R175H | SNV | 1.05% | 1.05% | 0.39% | 0.49% | 0.32% | ND | 0.20% | ND |
|  |  | TP53 | p.R248Q | SNV | 0.94% | 0.31% | 0.59% | 0.10% | 0.31% | ND | 0.14% | ND |
|  |  | TP53 | p.R273H | SNV | 1.06% | 0.91% | 0.56% | 0.62% | 0.26% | ND | 0.16% | ND |
