# Supplementary material for: Analytical performance evaluation of a commercial next generation sequencing liquid biopsy platform using plasma ctDNA, reference standards, and synthetic serial dilution samples derived from normal plasma
Source: BMC Cancer. 2020 Oct 1;20:945. doi: 10.1186/s12885-020-07445-5 (PMC7528227; doi:10.1186/s12885-020-07445-5)
Supplement: Supplementary file 7 — Additional file 7: Supplementary Table S4. Avenio Targeted ctDNA kit sensitivity (SNV/INDEL) [file 12885_2020_7445_MOESM7_ESM.docx]

**Supplementary Table S4:** Avenio Targeted ctDNA kit sensitivity (SNV/INDEL)

|  |  |  |  |  | 2.5% AF | | 0.5% AF | |
| --- | --- | --- | --- | --- | --- | --- | --- | --- |
| Gene Name | Cosmic ID | Mutation | AA change | TYPE | 10 ng | 20 ng | 10 ng | 20 ng |
| ALK | COSM144250 | c.3604G>A | p.G1202R | SNV | 2.58% | 3.04% | 0.92% | 0.54% |
| ALK | COSM28055 | c.3522C>A | p.F1174L | SNV | 2.64% | 1.84% | 0.71% | 0.62% |
| BRAF | COSM476 | c.1799T>A | p.V600E | SNV | 2.13% | 2.08% | 0.73% | 0.64% |
| EGFR | COSM6240 | c.2369C>T | p.T790M | SNV | 2.32% | 1.44% | 0.56% | 0.47% |
| EGFR | COSM6224 | c.2573T>G | p.L858R | SNV | 2.20% | 2.67% | ND | 0.29% |
| EGFR | COSM12370 | c.2240_2257del18 | p.L747_P753>S | DEL | 2.83% | 4.30% | 0.36% | 0.40% |
| EGFR | COSM6256 | c.2254_2277del24 | p.S752_I759 del SPKANKEI | DEL | 2.10% | 2.50% | 0.31% | 0.35% |
| EGFR | COSM6223 | c.2235_2249del15 | p.E746_A750 del ELREA c | DEL | 2.80% | 3.10% | 0.47% | 0.63% |
| ERBB2 | COSM20959 | c.2324_2325ins12 | p.A775_G776 ins YVMA | INS | 1.90% | 1.90% | 0.32% | 0.12% |
| KIT | COSM1314 | c.2447A>T | p.D816V | SNV | 1.96% | 2.17% | 0.66% | 0.47% |
| KRAS | COSM516 | c.34G>T | p.G12C | SNV | 2.21% | 2.75% | 0.29% | 0.58% |
| KRAS | COSM521 | c.35G>A | p.G12D | SNV | 2.41% | 2.73% | 0.46% | 0.75% |
| KRAS | COSM554 | c.183A>C | p.Q61H | SNV | 2.36% | 1.96% | 0.43% | 0.51% |
| NRAS | COSM584 | c.182A>G | p.Q61R | SNV | 2.74% | 2.83% | 0.75% | 0.80% |
| **Analytical Sensitivity** | | | | | **100%** | **100%** | **93%** | **100%** |
