# Supplementary material for: Analytical performance evaluation of a commercial next generation sequencing liquid biopsy platform using plasma ctDNA, reference standards, and synthetic serial dilution samples derived from normal plasma
Source: BMC Cancer. 2020 Oct 1;20:945. doi: 10.1186/s12885-020-07445-5 (PMC7528227; doi:10.1186/s12885-020-07445-5)
Supplement: Supplementary file 8 — Additional file 8: Supplementary Table S5. INDEL Loci of interest for Avenio Expanded kit [file 12885_2020_7445_MOESM8_ESM.docx]

**Supplementary Table S5:** INDEL Loci of interest for Avenio Expanded kit

| INDEL Loci of interest for Avenio Expanded kit | | | |
| --- | --- | --- | --- |
| #CHROM | POS | REF | ALT |
| chr10 | 87957958 | T | TA |
| chr10 | 87933146 | AC | A |
| chr10 | 87957959 | A | AC |
| chr10 | 87957960 | C | CA |
| chr10 | 87961041 | GTACT | G |
| chr10 | 87961054 | C | CA |
| chr17 | 39724728 | A | AGCATACGTGATG |
| chr17 | 39723965 | GTTGAGGGAAAACACA | G |
| chr17 | 39724748 | T | TGGGCTCCCC |
| chr17 | 39724749 | G | GGGCTCCCCA |
| chr17 | 39724749 | G | GGGCTCCCCT |
| chr2 | 29222405 | G | GCGT |
| chr3 | 179234361 | C | CA |
| chr3 | 41224642 | CCTT | C |
| chr4 | 54727434 | CAGTGGAAGGTTGTTG | C |
| chr4 | 54727434 | CAGTGGA | C |
| chr4 | 54727437 | TGGAAGG | T |
| chr4 | 54727442 | GGTT | G |
| chr4 | 54727495 | TTCCTTATGA | T |
| chr4 | 54727500 | TATG | T |
| chr5 | 112839514 | TAAAAG | T |
| chr5 | 112839548 | TC | T |
| chr5 | 112839826 | GT | G |
| chr5 | 112839978 | AAGAG | A |
| chr7 | 55174772 | GGAATTAAGAGAAGCA | G |
| chr7 | 55181319 | C | CGGT |
| chr7 | 55174749 | G | GTTAAAATTCCCGTCGCTA |
| chr7 | 55174751 | T | TAAAATTCCCGTCGCTATC |
| chr7 | 55174755 | A | ATTCCCGTCGCTATCAAGG |
| chr7 | 55174768 | TCAAGGAATTAAGAGAAGC | T |
| chr7 | 55174769 | CAAGGAATTAAGAGAA | C |
| chr7 | 55174770 | AAGGAATTAAGAGAAG | A |
| chr7 | 55174770 | AAGGAATTAAGAG | A |
| chr7 | 55174770 | AAGG | A |
| chr7 | 55174771 | AGGAATTAAGAGAAGCAAC | A |
| chr7 | 55174771 | AGGAATTAAGAGAAGC | A |
| chr7 | 55174772 | GGAATTAAGAGAAGCAACATCT | G |
| chr7 | 55174772 | GGAATTAAGAGAAGCAACA | G |
| chr7 | 55174772 | GGAATTAAGA | G |
| chr7 | 55174773 | GAATTAAGAGAAGCAACATCTCCGA | G |
| chr7 | 55174773 | GAATTAAGAGAAGCAACAT | G |
| chr7 | 55174773 | GAATTAAGAGAAGCAA | G |
| chr7 | 55174774 | AATTAAGAGAAGCAACATCTCC | A |
| chr7 | 55174774 | AATTAAGAGAAGCAACATC | A |
| chr7 | 55174774 | AATTAAGAGAAGCAAC | A |
| chr7 | 55174776 | TTAAGAGAAGCAACATCTC | T |
| chr7 | 55174776 | TTAAGAGAAGCAA | T |
| chr7 | 55174781 | AGAAGCAACATCTCCGAAAGCCAACAAG | A |
| chr7 | 55174782 | GAAGCAACATCTCCGA | G |
| chr7 | 55174786 | CAACATCTCCGAAAGCCAACAAGGA | C |
| chr7 | 55174789 | CATCTCCGAAAGCCAACAAGGAAAT | C |
| chr7 | 55174790 | ATCTCCGAAAGCCAACAAGGAAATC | A |
| chr7 | 55174793 | TCCGAAAGCCAACAAGGAAATC | T |
| chr7 | 55181305 | A | ATGGCCACGC |
| chr7 | 55181305 | A | ATGGCCAGCG |
| chr7 | 55181309 | C | CCAGCGTGGA |
| chr7 | 55181312 | G | GCGTGGGGGT |
| chr7 | 55181317 | G | GACAACCCCC |
| chr7 | 55181317 | G | GGTT |
| chr7 | 55181319 | C | CGGCACA |
| chr7 | 55181319 | C | CGGGTTT |
| chr7 | 55181320 | A | AACCCCC |
| chr7 | 55181323 | C | CCCCACG |
| chr7 | 55181324 | C | CCCACGT |
| chr7 | 55181324 | C | CCCA |
| chr7 | 55181325 | C | CCACGTG |
| chr7 | 55181381 | AG | A |
| chr7 | 55181456 | AC | A |
| chr7 | 116771825 | CTCTT | C |
| chr7 | 116771833 | TTCTC | T |
| chr7 | 116771986 | GAAGGT | G |
| chr7 | 116771988 | AG | A |
| chr7 | 116771989 | GGT | G |
| chr7 | 140753333 | TTCA | T |
| chr9 | 132905669 | CCT | C |
